# The era of cryptic exons: implications for ALS-FTD

**DOI:** 10.1186/s13024-023-00608-5

**Published:** 2023-03-15

**Authors:** Puja R. Mehta, Anna-Leigh Brown, Michael E. Ward, Pietro Fratta

**Affiliations:** 1 Department of Neuromuscular Diseases, UCL Queen Square Institute of Neurology, UCL Queen Square Motor Neuron Disease Centre, London, WC1N 3BG UK; 2 National Institute of Neurological Disorders and Stroke, NIH, Bethesda, MD USA

**Keywords:** Motor neuron disease, Amyotrophic lateral sclerosis, Frontotemporal dementia, TDP-43 proteinopathies, Cryptic exons, Splicing, Biomarkers, Therapeutics, UNC13A, STMN2

## Abstract

TDP-43 is an RNA-binding protein with a crucial nuclear role in splicing, and mislocalises from the nucleus to the cytoplasm in a range of neurodegenerative disorders. TDP-43 proteinopathy spans a spectrum of incurable, heterogeneous, and increasingly prevalent neurodegenerative diseases, including the amyotrophic lateral sclerosis and frontotemporal dementia disease spectrum and a significant fraction of Alzheimer’s disease. There are currently no directed disease-modifying therapies for TDP-43 proteinopathies, and no way to distinguish who is affected before death. It is now clear that TDP-43 proteinopathy leads to a number of molecular changes, including the de-repression and inclusion of cryptic exons. Importantly, some of these cryptic exons lead to the loss of crucial neuronal proteins and have been shown to be key pathogenic players in disease pathogenesis (e.g.*, STMN2*), as well as being able to modify disease progression (e.g.*, UNC13A*). Thus, these aberrant splicing events make promising novel therapeutic targets to restore functional gene expression. Moreover, presence of these cryptic exons is highly specific to patients and areas of the brain affected by TDP-43 proteinopathy, offering the potential to develop biomarkers for early detection and stratification of patients. In summary, the discovery of cryptic exons gives hope for novel diagnostics and therapeutics on the horizon for TDP-43 proteinopathies.

## Background

‘TDP-43 proteinopathy’ refers to human diseases pathologically defined by cellular nuclear-to-cytoplasmic mislocalisation and aggregation of TAR DNA-binding protein-43 (TDP-43), an RNA-binding protein with a crucial role in RNA metabolism and splicing. TDP-43 proteinopathy encompasses a wide spectrum of progressive neurodegenerative diseases and phenotypes. It is a key feature in ~ 97% of people with amyotrophic lateral sclerosis (ALS) and ~ 45% of people with frontotemporal dementia (FTD), a seminal discovery made in 2006 [1, 2]. Subsequently, TDP-43 proteinopathy has been found as a co-pathology in a large fraction of Alzheimer’s disease, as well as inclusion body myositis and Paget disease of bone [3–5]. Furthermore, TDP-43 mislocalisation can be trigged by traumatic stimuli, such as physical stress or brain injury, which are themselves increasingly recognised as risk factors for a number of the above described neurodegenerative disorders [6–9].

Common to TDP-43 proteinopathies is a lack of directed disease-modifying therapies, and an inability to confirm and stratify individuals that have TDP-43 pathology ante-mortem. The disease that is most frequently characterised by TDP-43 mislocalisation is ALS, which is a rapidly progressive paralysing illness with eventual death on average 3 years from symptom onset [10]. Up to 15% of people with ALS can experience associated cognitive, language, and behavioural deficits, consistent with a diagnosis of FTD, comprising the ALS-FTD spectrum [11]. Pathogenic mutations in *TARDBP*, the gene which encodes TDP-43, are rare, occurring in < 1% of ALS cases, but underscore TDP-43’s central role in disease pathogenesis [12–14]. Altered subcellular localisation of TDP-43 is relevant – either via cytoplasmic toxic gain of function, nuclear loss of function, or both. However, the precise molecular mechanisms downstream of TDP-43 mislocalisation remain incompletely understood.

**Fig. 1:**
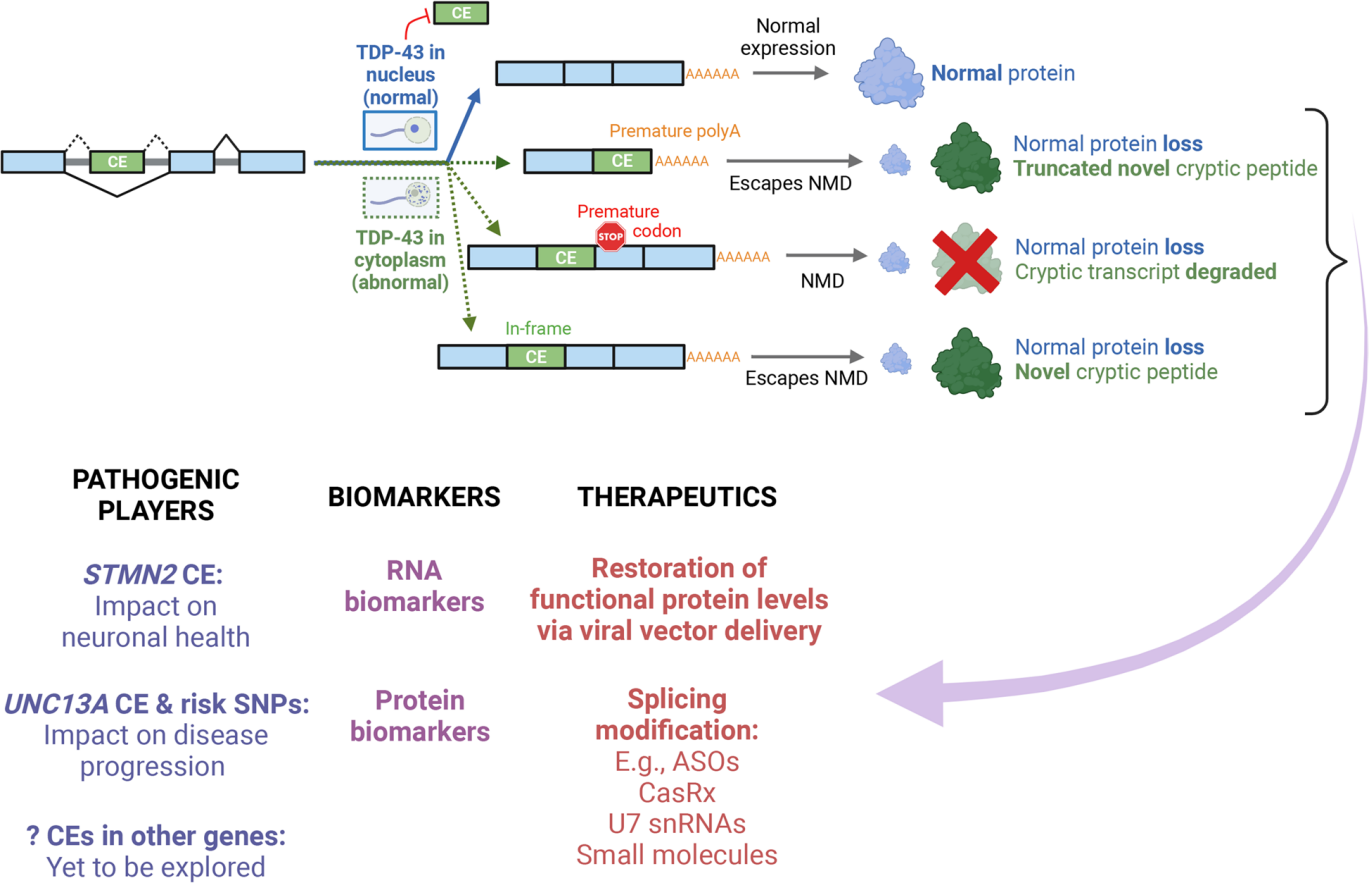
Schematic summarising how cryptic exons (CEs) arise in the context of TDP-43 mislocalisation from the nucleus to the cytoplasm (owing to de-repression of splicing in intronic regions), possible downstream consequences (loss of functional protein, nonsense-mediated decay (NMD) of the cryptic-containing transcript, or translation of a novel cryptic peptide), and opportunities that CEs provide for better understanding disease mechanisms (*STMN2, UNC13A,* other genes yet to be explored), biomarker development (RNA and protein biomarkers), and therapeutics (via restoration of protein levels, or splicing modification)

In this review, we will focus on a key ‘on-off’ consequence of nuclear TDP-43 loss of function, which has emerged over recent years – the occurrence of splicing defects leading to the erroneous inclusion of intronic sequences into mature mRNA, forming so-called ‘cryptic exons’ [15]. With a spotlight on ALS-FTD, we will review their relevance to disease pathogenesis, and discuss exciting and emerging opportunities on the horizon for biomarker development and therapeutics in the field of TDP-43 proteinopathies (Fig. 1).

### TDP-43 depletion leads to de-repression and inclusion of cryptic exons

TDP-43 was identified in 1995 as a suppressor of HIV gene expression. It was later discovered that TDP-43 expression promotes skipping of exon 9 in the *CFTR* gene, first realising TDP-43’s role as an RNA-binding protein and regulator of alternative splicing [16]. Since then, an increasing number of genes have been identified for which the splicing of annotated, conserved exons is regulated by TDP-43; as such, altered splicing in *POLDIP3*, *SORT1*, and *PFKP* have all been used as reliable readouts for nuclear TDP-43 function in models with TDP-43 knockout, overexpression, and for studying TDP-43 ALS-causing mutations [17–27].

TDP-43’s consensus binding motif comprises “UG”-rich dinucleotide repeats. High-throughput sequencing, combined with cross-linking immunoprecipitation experiments to discover direct TDP-43 mRNA targets, revealed that the majority of TDP-43’s binding sites in pre-RNAs were in introns [26, 27]. A number of these intronic binding sites were later revealed to be sites where TDP-43 binding acts as a splicing repressor. When, as a result of TDP-43 depletion, non-conserved, intronic sequences are erroneously included in mature RNA, they are called cryptic exons [15]. Importantly, these cryptic exons can be found in patient tissues from people affected by ALS and FTD, Alzheimer’s disease, and inclusion body myositis [15, 23, 28–34]. While TDP-43 regulation of annotated splicing leads to shifts in the ratio of known isoforms, TDP-43 cryptic splicing produces novel RNA isoforms. These changes include novel cassette exons, skipping of canonical splicing products, 3′ or 5′ extensions of annotated exons, novel transcription start sites, and novel poly-adenylation events [15, 35].

What are the possible consequences that result from the insertion of novel non-conserved sequences in mRNA (Fig. 1)? Most cryptic exons are predicted to lead to destabilisation and degradation of mRNA, thereby resulting in a reduction in functional levels of the corresponding protein. This occurs primarily through nonsense-mediated decay (NMD) due to introduction of frameshifts and premature termination codons [32, 36]. Conceptually, such cryptic exons could lead to deleterious downstream physiological consequences if they reside in transcripts encoding critical proteins. Occasionally, cryptic exons lead to an in-frame change to the nucleotide sequence with no premature stop codons, thereby escaping NMD and resulting in a predicted translation of novel cryptic peptides. Alternatively, they can result in premature polyadenylation, potentially leading to truncated proteins. It is conceivable that these proteins resulting from mis-spliced RNA could acquire gain of function changes in their biology, be toxic, or serve as detectable biomarkers for monitoring of disease activity, or for stratification of subtypes in ALS-FTD and other neurodegenerative disorders.

### Relevance to disease

#### Impact on neuronal health – STMN2

In 2019, a breakthrough discovery from two independent groups found that TDP-43 loss of function leads to the inclusion of a cryptic exon within the *STMN2* gene [23, 30]*.* This gene encodes for stathmin-2, a microtubule-associated protein needed for axonal growth and repair in neurons. Occurring after exon 1 and containing a stop codon and polyadenylation site, the cryptic exon leads to premature transcript termination and predicted formation of a truncated non-functional protein. Using in vitro neuronal models of TDP-43 depletion via knockdown, induction of TDP-43 mislocalisation, and pathological loss of function TDP-43 mutations known to cause ALS, the research groups demonstrated a resultant dramatic reduction in levels of mature *STMN2* mRNA and functional protein. Crucially, the *STMN2* cryptic exon was confirmed to be detectable also in ALS-FTD brains and spinal cords where TDP-43 pathology is present and specifically in patient post-mortem neurons with TDP-43 pathology isolated by laser capture or nuclear sorting [30–32, 37]. Without TDP-43, motor neuron axons were unable to regenerate after an axotomy. Remarkably, they were able to rescue axonal regrowth by restoring levels of STMN2 protein, thereby demonstrating that STMN2 is critical to the health of motor neurons, and that its TDP-43 dependent loss can be a key pathogenic player in ALS. Following this pivotal discovery, the hunt for other cryptic exons that might contribute to or exacerbate ALS pathology continued.

#### Impact on ALS disease progression – UNC13A

*UNC13A* is one of the top genetic risk factors for ALS, first reported in a genome-wide association study in 2009 [38]. UNC13A protein plays an important role in neurotransmitter release and synaptic transmission, and mice that lack this gene (called *Munc13–1* in mice) have synaptic impairments and die soon after birth [39]. In humans, rare cases with homozygous nonsense mutations in *UNC13A* result in fatal microcephaly, cortical hyperexcitability, and myasthenia [40]. Single nuclear polymorphisms (SNPs) in *UNC13A* have been shown to increase risk of ALS and also shorten survival in patients. However, the underlying mechanism by which these *UNC13A* variants increase ALS risk was not known until earlier this year when, along with the Gitler and Petrucelli groups, we independently found that TDP-43 depletion resulted in inclusion of a cryptic exon between exons 20 and 21 of *UNC13A*, leading to reduced expression. Similarly to the *STMN2* cryptic exon, this event can also be detected in post-mortem brain and spinal cord tissue from patients with ALS-FTD, exclusively where TDP-43 pathology is present, but not in healthy controls.

Intriguingly, two of the associated *UNC13A* SNPs, rs12608932 (A > C) and rs12973192 (C > G), were found to be located within the intron containing the cryptic exon, with the former SNP being located within the cryptic exon region itself. Especially given the large size of the *UNC13A* gene (~ 87 kb), the proximity of the SNPs to the cryptic exon led us to hypothesise a direct link between the SNPs and *UNC13A* cryptic splicing. Given that the cryptic exon is not present in RNA-sequencing data from healthy controls, even when homozygous for the risk SNPs (approximately 10% of Caucasians are homozygous for both risk SNPs), they seem not to be sufficient to cause cryptic exon inclusion on their own. Thus, it was hypothesised that rather than increasing ALS or FTD risk directly, the SNPs act by potentiating the expression of a cryptic exon which is dependent on TDP-43 depletion.

Indeed, the risk SNPs enhance cryptic splicing and alter direct TDP-43 binding, thereby reducing its ability to physiologically repress cryptic exon inclusion. In keeping with this, patients homozygous for either SNP had more *UNC13A* cryptic exon-containing transcripts in a dose-responsive manner compared to heterozygotes and non-carriers. This discovery provides a biological explanation for how presence of the *UNC13A* risk SNPs in ALS-FTD patients with TDP-43 pathology modifies disease outcomes, negatively affecting survival from symptom onset [32, 33, 41–47]. Further work needs to be done to understand the biology connecting the above molecular events and disease progression, by specifically investigating the functional consequences of synaptic dysfunction associated with TDP-43 and UNC13A loss.

### Future directions

#### Opportunities for novel biomarkers

Biomarkers for early diagnosis, disease stratification of subtypes and clinical stages, prognostication, and monitoring treatment response are vitally important, both for clinical trials and patient care. Fluid biomarkers of general neurodegeneration exist, most notably serum neurofilament light chain, cerebrospinal fluid (CSF) phosphorylated neurofilament heavy chain, and urinary p75^ECD^ [48–50]. However, biomarkers specific for ALS-FTD and other TDP-43 proteinopathies are not currently available, nor are there readouts of TDP-43 function in living patients.

Unlike canonical TDP-43 regulated splicing, the ‘on-off’ expression of cryptic exons only under TDP-43 nuclear loss of function makes them potential potent biomarker candidates for TDP-43 pathology. This proof of concept was first shown with the *STMN2* cryptic exon in post-mortem frontal cortex samples, in which its presence was able to discriminate between patients with FTD-TDP, progressive supranuclear palsy, and healthy control participants [31].

A key future direction will be to exploit this characteristic to develop methods that detect TDP-43 pathology ante-mortem in patient biofluids, such as in blood, CSF, urine, or tissue. Assays could involve either direct RNA detection of the cryptic exon itself, or detection of novel cryptic peptides for transcripts that escape NMD. An ideal biomarker would have a reproducibly high and linear correlation with disease activity, high sensitivity and specificity, stability throughout the day, and easy access to obtain a sample [51].

With regards to biomarker detection of RNA, methods have been developed that can sensitively detect circulating RNA in the blood of individuals with Alzheimer’s disease that reflect disease-specific neural transcriptome changes [52]. However, there are challenges. For example, most cryptic exon-containing transcripts, such as for *UNC13A*, undergo NMD and are therefore not stable. Moreover, if transcripts are at low levels, highly sensitive tools would be required. Detection of a panel of relevant cryptic exons deemed to be pathogenic players could theoretically increase sensitivity.

For cryptic exons that produce cryptic peptides, specific antibodies could be designed to detect these via immunohistochemistry, or ELISA and SIMOA-based assays. This has its own challenges, whether it be due to NMD of cryptic-containing transcripts, low expression, or instability of the peptides. For *STMN2*, even though the cryptic-containing transcript is highly expressed and escapes NMD, attempts to detect the putative 16 amino acid cryptic peptide have not yet been successful. However, antibody generation to specifically detect pathological proteins is evolving, including in the field of ALS-FTD, with antibodies now available to detect dipeptide repeats that occur in the *C9orf72* subtype of ALS-FTD. These have not only furthered our understanding of disease mechanisms, but are also being utilised to assess therapies [53–56].

Excitingly, two new pre-print publications this year have independently reported the detection of cryptic peptides in the CSF of patients with ALS [57, 58]. Irwin et al. demonstrated that a newly characterised monoclonal antibody, specific to a TDP-43-dependent cryptic epitope encoded by the cryptic exon found in *HDGFL2,* detects the cryptic peptide in *C9orf72*-associated ALS. Strikingly, this includes pre-symptomatic mutation carriers [57]. In Seddighi et al., our groups first demonstrated the presence of de novo cryptic peptides in iPSC-derived neurons with TDP-43 knockdown, and then used a novel targeted proteomics assay to confirm the presence of cryptic peptides in CSF of patients with ALS-FTD [58]. Further work will be needed to assess the specificity and sensitivity of cryptic peptides as biomarkers, but, taken together, these discoveries are encouraging steps towards facilitating earlier diagnosis of ALS, and also providing a way of measuring target engagement in clinical trials for new therapies aimed at restoring TDP-43 function.

Given the cell-type specificity of TDP-43 controlled cryptic exons, it is also possible that biomarkers towards cryptic exons could be designed to assess the flow of TDP-43 proteinopathy across the nervous tissue [25, 35, 59, 60]. While TDP-43 proteinopathy in cortical and motor neurons has received much attention, TDP-43 proteinopathy is also present in glia and Schwann cells [2, 60–63]. Indeed, TDP-43 depletion in mouse Schwann cells showed that TDP-43 regulates the inclusion of a cell-type specific cryptic exon in *Neurofascin* in these cells, highlighting how, if certain TDP-43 cryptic exons are expressed only by given cells, they could allow tracing of TDP-43 proteinopathy. Furthermore, recent work showing that pTDP-43 and loss of nuclear TDP-43 can be detected years before ALS disease presentation in central nervous system and non-central nervous system tissue suggests that detection of TDP-43 cryptic exons could possibly pre-date disease onset [64, 65].

Another interesting avenue is the use of model systems to determine the timing of the emergence of cryptic exon inclusion events. For instance, studies assessing the presence of cryptic exons at different levels of TDP-43 knockdown in vitro, or in patients with or post-mortem tissue from varying clinical stages of ALS-FTD, could allow curation of a database reflecting which cryptic exons appear early and late in disease. Further analysis of this could provide a greater mechanistic understanding of cryptic exon biology and what factors make certain genes more sensitive and susceptible to the inclusion of cryptic exons with TDP-43 loss. Such a stratification may also have implications for biomarker studies and early diagnosis and stratification of people with ALS-FTD.

Further work is necessary to utilise the specificity of cryptic exons to develop appropriate biomarkers, which could be transformative to the field of ALS-FTD and other TDP-43 proteinopathies. Particularly with new therapies on the horizon for neurodegenerative diseases, early diagnosis of individuals who are or will become affected and a readout for treatment response will be critical in halting the disease process at an early enough stage to pre-empt the point of no return in the ALS disease cascade [66].

#### Opportunities for therapeutics

The discovery that certain cryptic exons, and potentially more that remain to be explored, underpin the disease process in ALS – either by impairing neuronal health, increasing ALS risk, or worsening disease progression – makes them exciting novel therapeutic targets. If early diagnosis can be achieved, the hope would be to restore expression of critical proteins to normal functional levels, or use splicing therapies to modulate and prevent aberrant splicing from occurring altogether in TDP-43 proteinopathies.

The *STMN2* cryptic exon leads to a reduction in *STMN2* expression. Thus, one strategy would be to restore normal STMN2 levels using a viral vector containing the correct DNA sequence, such as via adeno-associated viral (AAV) or lentiviral delivery. This proof of concept was shown when transduction of TDP-43 depleted iPSC-derived motor neurons with lentivirus carrying *STMN2* restored STMN2 levels and also, importantly, restored axonal regeneration after axotomy [30]. It was also recently shown in mice deficient of *Stmn2* (*Stmn2*^*−/−*^*)* that the introduction of the human *STMN2* gene was able to rescue motor deficits [67]. However, translating this method to humans poses challenges, including discerning what the target level should be and at what point to treat in order to avoid missing the effective therapeutic window but also avoid overexpression and potential gain of function toxicity, as was observed in spinal muscular atrophy (SMA) studies as a result of *SMN* overexpression [68]. Moreover, it would be important that such therapies are directed to the correct anatomical site with appropriate tissue and cell-type specificity to prevent off-target effects, and also that any harmful immunogenic responses to the viral vector itself are avoided [69]. Another obstacle would be the limitation in packaging capacity of the viral vector and therefore the size of the gene needing to be restored. As such, *UNC13A* would be too large to be introduced via this methodology, as opposed to *STMN2*, which would theoretically be more amenable*.*

Other important gene-therapy strategies involve the prevention of erroneous RNA splicing. Compared to the above described gene delivery methods, these approaches aim to correct endogenous splicing and therefore avoid problems deriving from overexpression and toxicity. One method is via antisense oligonucleotide therapies (ASOs), which are short synthetic DNA sequences that are designed to selectively bind to pre-mRNA in cells. ASOs have come to the forefront as a new tool for numerous neurodegenerative diseases, including toxic gain of function gene-specific ALS subtypes, where they have been used to degrade mutant transcripts. ASOs designed to reduce protein produced from mutant *SOD1* and *C9orf72* have been tested in ALS clinical trials (BIIB067 and BIIB078, respectively). The former showed very encouraging results, but failed to reach significance in achieving primary clinical endpoints in a phase 3 trial, and the latter phase 1 trial showed no difference from placebo for clinical endpoints and in fact trended towards greater decline at the higher dose. These results highlight some of the caveats of ASO therapies [70, 71]. ASOs can also be used to directly introduce splicing modulation, and this has been used to improve functional splicing in disorders, such as SMA, or to limit the effect of deleterious mutations by skipping specific exons, such as in Duchenne muscular dystrophy (DMD) [72–75]. One drawback of ASO therapies is the need for repeated dosing for a maintained response, which is particularly undesirable for patients if the delivery method is invasive, such as via intrathecal administration.

Other effective splice switching strategies include systems that target pre-mRNA, but are stably expressed in the long-term, thereby preventing the need for repeated dosages, whilst also aiming to circumvent the above concerns regarding overexpression. These include CRISPR-Cas ribonuclease programming, such as via RfxCas13d (also named CasRx, from *Ruminococcus flavefaciens*), and using modified uridine-rich small nuclear RNA (snRNA) gene therapy, such as via U7 snRNA delivery, which has shown promise in DMD, and has entered its first clinical trial [76–78]. In addition to the above benefits, both methods involve relatively small-sized effectors, such that they can be packaged into a viral vector, and theoretically may even allow for multiple cryptic exons to be targeted in parallel.

Finally, small molecule delivery has been developed as a further tool to alter splicing. A clear benefit of this therapeutic platform is the ability to deliver the treatment to patients orally. The first small molecule splicing modifier to be approved was Risdiplam for oral delivery in SMA. It was shown to increase SMN protein in the central nervous system and peripheral tissues in mice, and to restore functional SMN protein in patients with SMA [79–82].

Homing in on correcting cryptic exon-related mis-splicing, a phase I clinical trial is planned to assess an ASO designed to restore STMN2 levels [83]. Commonly in the field of therapeutics, mouse models are used prior to testing in humans; however, human cryptic exons have been found to share very little overlap with mouse cryptic exons, making this a problem [15]. The cryptic exons in both *STMN2* and *UNC13A* that have been reported in humans are not present in mice. In fact, and interestingly, different *UNC13A* cryptic exons are present in mice [15]. Therefore, in vivo models are needed to test splicing therapies that target specific cryptic exons. One possibility is the development of humanised mouse models containing the relevant human cryptic exons [84, 85]. Another issue arising from the use of human iPSC models alone to study therapeutics in the field of cryptic exons is that cryptic exon signatures in TDP-43 depletion have been shown to be highly variable between cell types [25, 35]. Whilst there was some commonality between stem cells, neurons, and myocytes, most non-conserved cryptic exons were cell-type specific. Thus, TDP-43 loss of function may impair cell-type specific pathways, which has mechanistic and treatment-evaluation implications that would need to be considered. With the development of better models for assessing human-relevant cryptic exons, there is significant promise for novel therapies on the horizon.

Whilst blocking TDP-43 cryptic exons to restore gene expression appears to have a relatively clear mechanistic benefit, it remains to be seen how many and which cryptic exons need to be targeted for an effective therapy. Future experiments may seek to determine which cryptic exon inclusion events are pathologically relevant, impacting on a biological pathway that contributes to disease, as this may impact the development of therapies and potential ways to restore the expression of multiple mRNA targets, as opposed to one at a time.

## Conclusions

The past decade has seen leaps forward for people with gene-specific ALS-FTD, now with gene-based therapies coming into play. However, these would not benefit people with sporadic disease, making up the majority of patients with ALS-FTD, or those that have different pathogenic genes that are not amenable to treatment with available gene-based therapies. Given that almost all patients with ALS have TDP-43 pathology, the recent breakthroughs revealing that TDP-43 dependent mis-splicing of mRNA plays a major role in disease pathogenesis provides a fresh injection of hope into the field, not only for the ability to better understand molecular mechanisms, but also for the transformative potential with regard to biomarker development and therapeutics, desperately needed for patients living with this fatal disease. Important focuses going forward will be to establish which other cryptic exons, if any, are pathogenic players, and for the knowledge gained about cryptic exons to be transferred to and explored in other TDP-43 proteinopathies.

## Data Availability

Not applicable.

## Abbreviations

ALS: Amyotrophic lateral sclerosis
FTD: Frontotemporal dementia
CE: Cryptic exon
NMD: Nonsense-mediated decay
ASO: Antisense oligonucleotide
snRNA: Small nuclear RNA
SMA: Spinal muscular atrophy
DMD: Duchenne muscular dystrophy
AAV: Adeno-associated virus
SNP: Single nucleotide polymorphism

## References

[1] Neumann M, Sampathu DM, Kwong LK, Truax AC, Micsenyi MC, Chou TT, Bruce J, Schuck T, Grossman M, Clark CM (2006). Ubiquitinated TDP-43 in frontotemporal lobar degeneration and amyotrophic lateral sclerosis. Science.

[2] Arai T, Hasegawa M, Akiyama H, Ikeda K, Nonaka T, Mori H, Mann D, Tsuchiya K, Yoshida M, Hashizume Y, Oda T (2006). TDP-43 is a component of ubiquitin-positive tau-negative inclusions in frontotemporal lobar degeneration and amyotrophic lateral sclerosis. Biochem Biophys Res Commun.

[3] Higashi S, Iseki E, Yamamoto R, Minegishi M, Hino H, Fujisawa K, Togo T, Katsuse O, Uchikado H, Furukawa Y (2007). Concurrence of TDP-43, tau and alpha-synuclein pathology in brains of Alzheimer's disease and dementia with Lewy bodies. Brain Res.

[4] de Boer EMJ, Orie VK, Williams T, Baker MR, De Oliveira HM, Polvikoski T, Silsby M, Menon P, van den Bos M, Halliday GM (2020). TDP-43 proteinopathies: a new wave of neurodegenerative diseases. J Neurol Neurosurg Psychiatry.

[5] Neumann M, Mackenzie IR, Cairns NJ, Boyer PJ, Markesbery WR, Smith CD, Taylor JP, Kretzschmar HA, Kimonis VE, Forman MS (2007). TDP-43 in the ubiquitin pathology of frontotemporal dementia with VCP gene mutations. J Neuropathol Exp Neurol.

[6] Moisse K, Volkening K, Leystra-Lantz C, Welch I, Hill T, Strong MJ (2009). Divergent patterns of cytosolic TDP-43 and neuronal progranulin expression following axotomy: implications for TDP-43 in the physiological response to neuronal injury. Brain Res.

[7] Wiesner D, Tar L, Linkus B, Chandrasekar A, Olde Heuvel F, Dupuis L, Tsao W, Wong PC, Ludolph A, Roselli F (2018). Reversible induction of TDP-43 granules in cortical neurons after traumatic injury. Exp Neurol.

[8] Wang HK, Lee YC, Huang CY, Liliang PC, Lu K, Chen HJ, Li YC, Tsai KJ (2015). Traumatic brain injury causes frontotemporal dementia and TDP-43 proteolysis. Neuroscience.

[9] Gao F, Hu M, Zhang J, Hashem J, Chen C (2022). TDP-43 drives synaptic and cognitive deterioration following traumatic brain injury. Acta Neuropathol.

[10] del Aguila MA, Longstreth WT, McGuire V, Koepsell TD, van Belle G (2003). Prognosis in amyotrophic lateral sclerosis: a population-based study. Neurology.

[11] Ringholz GM, Appel SH, Bradshaw M, Cooke NA, Mosnik DM, Schulz PE (2005). Prevalence and patterns of cognitive impairment in sporadic ALS. Neurology.

[12] Mackenzie IR, Rademakers R, Neumann M (2010). TDP-43 and FUS in amyotrophic lateral sclerosis and frontotemporal dementia. Lancet Neurol.

[13] Kabashi E, Valdmanis PN, Dion P, Spiegelman D, McConkey BJ, Vande Velde C, Bouchard JP, Lacomblez L, Pochigaeva K, Salachas F (2008). TARDBP mutations in individuals with sporadic and familial amyotrophic lateral sclerosis. Nat Genet.

[14] Sreedharan J, Blair IP, Tripathi VB, Hu X, Vance C, Rogelj B, Ackerley S, Durnall JC, Williams KL, Buratti E (2008). TDP-43 mutations in familial and sporadic amyotrophic lateral sclerosis. Science.

[15] Ling JP, Pletnikova O, Troncoso JC, Wong PC (2015). TDP-43 repression of nonconserved cryptic exons is compromised in ALS-FTD. Science.

[16] Buratti E, Baralle FE (2001). Characterization and functional implications of the RNA binding properties of nuclear factor TDP-43, a novel splicing regulator of CFTR exon 9. J Biol Chem.

[17] Buratti E, De Conti L, Stuani C, Romano M, Baralle M, Baralle F (2010). Nuclear factor TDP-43 can affect selected microRNA levels. FEBS J.

[18] Prudencio M, Jansen-West KR, Lee WC, Gendron TF, Zhang YJ, Xu YF, Gass J, Stuani C, Stetler C, Rademakers R (2012). Misregulation of human sortilin splicing leads to the generation of a nonfunctional progranulin receptor. Proc Natl Acad Sci U S A.

[19] Fiesel FC, Weber SS, Supper J, Zell A, Kahle PJ (2012). TDP-43 regulates global translational yield by splicing of exon junction complex component SKAR. Nucleic Acids Res.

[20] Shiga A, Ishihara T, Miyashita A, Kuwabara M, Kato T, Watanabe N, Yamahira A, Kondo C, Yokoseki A, Takahashi M (2012). Alteration of POLDIP3 splicing associated with loss of function of TDP-43 in tissues affected with ALS. PLoS One.

[21] Prpar Mihevc S, Baralle M, Buratti E, Rogelj B (2016). TDP-43 aggregation mirrors TDP-43 knockdown, affecting the expression levels of a common set of proteins. Sci Rep.

[22] Mohagheghi F, Prudencio M, Stuani C, Cook C, Jansen-West K, Dickson DW, Petrucelli L, Buratti E (2016). TDP-43 functions within a network of hnRNP proteins to inhibit the production of a truncated human SORT1 receptor. Hum Mol Genet.

[23] Klim JR, Williams LA, Limone F, Guerra San Juan I, Davis-Dusenbery BN, Mordes DA, Burberry A, Steinbaugh MJ, Gamage KK, Kirchner R (2019). ALS-implicated protein TDP-43 sustains levels of STMN2, a mediator of motor neuron growth and repair. Nat Neurosci.

[24] Roczniak-Ferguson A, Ferguson SM (2019). Pleiotropic requirements for human TDP-43 in the regulation of cell and organelle homeostasis. Life Sci Alliance.

[25] Susnjar U, Skrabar N, Brown AL, Abbassi Y, Phatnani H, Consortium NA, Cortese A, Cereda C, Bugiardini E, Cardani R (2022). Cell environment shapes TDP-43 function with implications in neuronal and muscle disease. Commun Biol.

[26] Tollervey JR, Curk T, Rogelj B, Briese M, Cereda M, Kayikci M, Konig J, Hortobagyi T, Nishimura AL, Zupunski V (2011). Characterizing the RNA targets and position-dependent splicing regulation by TDP-43. Nat Neurosci.

[27] Polymenidou M, Lagier-Tourenne C, Hutt KR, Huelga SC, Moran J, Liang TY, Ling SC, Sun E, Wancewicz E, Mazur C (2011). Long pre-mRNA depletion and RNA missplicing contribute to neuronal vulnerability from loss of TDP-43. Nat Neurosci.

[28] Sun M, Bell W, LaClair KD, Ling JP, Han H, Kageyama Y, Pletnikova O, Troncoso JC, Wong PC, Chen LL (2017). Cryptic exon incorporation occurs in Alzheimer's brain lacking TDP-43 inclusion but exhibiting nuclear clearance of TDP-43. Acta Neuropathol.

[29] Torres P, Ramirez-Nunez O, Romero-Guevara R, Bares G, Granado-Serrano AB, Ayala V, Boada J, Fontdevila L, Povedano M, Sanchis D (2018). Cryptic exon splicing function of TARDBP interacts with autophagy in nervous tissue. Autophagy.

[30] Melamed Z, Lopez-Erauskin J, Baughn MW, Zhang O, Drenner K, Sun Y, Freyermuth F, McMahon MA, Beccari MS, Artates JW (2019). Premature polyadenylation-mediated loss of stathmin-2 is a hallmark of TDP-43-dependent neurodegeneration. Nat Neurosci.

[31] Prudencio M, Humphrey J, Pickles S, Brown AL, Hill SE, Kachergus JM, Shi J, Heckman MG, Spiegel MR, Cook C (2020). Truncated stathmin-2 is a marker of TDP-43 pathology in frontotemporal dementia. J Clin Invest.

[32] Brown AL, Wilkins OG, Keuss MJ, Hill SE, Zanovello M, Lee WC, Bampton A, Lee FCY, Masino L, Qi YA (2022). TDP-43 loss and ALS-risk SNPs drive mis-splicing and depletion of UNC13A. Nature.

[33] Ma XR, Prudencio M, Koike Y, Vatsavayai SC, Kim G, Harbinski F, Briner A, Rodriguez CM, Guo C, Akiyama T (2022). TDP-43 represses cryptic exon inclusion in the FTD-ALS gene UNC13A. Nature.

[34] Britson KA, Ling JP, Braunstein KE, Montagne JM, Kastenschmidt JM, Wilson A, Ikenaga C, Tsao W, Pinal-Fernandez I, Russell KA (2022). Loss of TDP-43 function and rimmed vacuoles persist after T cell depletion in a xenograft model of sporadic inclusion body myositis. Sci Transl Med.

[35] Jeong YH, Ling JP, Lin SZ, Donde AN, Braunstein KE, Majounie E, Traynor BJ, LaClair KD, Lloyd TE, Wong PC (2017). Tdp-43 cryptic exons are highly variable between cell types. Mol Neurodegener.

[36] Lykke-Andersen S, Jensen TH (2015). Nonsense-mediated mRNA decay: an intricate machinery that shapes transcriptomes. Nat Rev Mol Cell Biol.

[37] Liu EY, Russ J, Cali CP, Phan JM, Amlie-Wolf A, Lee EB (2019). Loss of nuclear TDP-43 is associated with Decondensation of LINE retrotransposons. Cell Rep.

[38] van Es MA, Veldink JH, Saris CG, Blauw HM, van Vught PW, Birve A, Lemmens R, Schelhaas HJ, Groen EJ, Huisman MH (2009). Genome-wide association study identifies 19p13.3 (UNC13A) and 9p21.2 as susceptibility loci for sporadic amyotrophic lateral sclerosis. Nat Genet.

[39] Augustin I, Rosenmund C, Sudhof TC, Brose N (1999). Munc13-1 is essential for fusion competence of glutamatergic synaptic vesicles. Nature.

[40] Engel AG, Selcen D, Shen XM, Milone M, Harper CM (2016). Loss of MUNC13-1 function causes microcephaly, cortical hyperexcitability, and fatal myasthenia. Neurol Genet.

[41] Diekstra FP, van Vught PW, van Rheenen W, Koppers M, Pasterkamp RJ, van Es MA, Schelhaas HJ, de Visser M, Robberecht W, Van Damme P (2012). UNC13A is a modifier of survival in amyotrophic lateral sclerosis. Neurobiol Aging.

[42] Chio A, Mora G, Restagno G, Brunetti M, Ossola I, Barberis M, Ferrucci L, Canosa A, Manera U, Moglia C (2013). UNC13A influences survival in Italian amyotrophic lateral sclerosis patients: a population-based study. Neurobiol Aging.

[43] Vidal-Taboada JM, Lopez-Lopez A, Salvado M, Lorenzo L, Garcia C, Mahy N, Rodriguez MJ, Gamez J (2015). UNC13A confers risk for sporadic ALS and influences survival in a Spanish cohort. J Neurol.

[44] Gaastra B, Shatunov A, Pulit S, Jones AR, Sproviero W, Gillett A, Chen Z, Kirby J, Fogh I, Powell JF (2016). Rare genetic variation in UNC13A may modify survival in amyotrophic lateral sclerosis. Amyotroph Lateral Scler Frontotemporal Degener.

[45] Yang B, Jiang H, Wang F, Li S, Wu C, Bao J, Zhu Y, Xu Z, Liu B, Ren H, Yang X (2019). UNC13A variant rs12608932 is associated with increased risk of amyotrophic lateral sclerosis and reduced patient survival: a meta-analysis. Neurol Sci.

[46] Placek K, Baer GM, Elman L, McCluskey L, Hennessy L, Ferraro PM, Lee EB, Lee VMY, Trojanowski JQ, Van Deerlin VM (2019). UNC13A polymorphism contributes to frontotemporal disease in sporadic amyotrophic lateral sclerosis. Neurobiol Aging.

[47] Tan HHG, Westeneng HJ, van der Burgh HK, van Es MA, Bakker LA, van Veenhuijzen K, van Eijk KR, van Eijk RPA, Veldink JH, van den Berg LH (2020). The distinct traits of the UNC13A polymorphism in amyotrophic lateral sclerosis. Ann Neurol.

[48] Shepheard SR, Wuu J, Cardoso M, Wiklendt L, Dinning PG, Chataway T, Schultz D, Benatar M, Rogers ML (2017). Urinary p75(ECD): a prognostic, disease progression, and pharmacodynamic biomarker in ALS. Neurology.

[49] Rossi D, Volanti P, Brambilla L, Colletti T, Spataro R, La Bella V (2018). CSF neurofilament proteins as diagnostic and prognostic biomarkers for amyotrophic lateral sclerosis. J Neurol.

[50] Verde F, Steinacker P, Weishaupt JH, Kassubek J, Oeckl P, Halbgebauer S, Tumani H, von Arnim CAF, Dorst J, Feneberg E (2019). Neurofilament light chain in serum for the diagnosis of amyotrophic lateral sclerosis. J Neurol Neurosurg Psychiatry.

[51] Henley SM, Bates GP, Tabrizi SJ (2005). Biomarkers for neurodegenerative diseases. Curr Opin Neurol.

[52] Koh W, Pan W, Gawad C, Fan HC, Kerchner GA, Wyss-Coray T, Blumenfeld YJ, El-Sayed YY, Quake SR (2014). Noninvasive in vivo monitoring of tissue-specific global gene expression in humans. Proc Natl Acad Sci U S A.

[53] Mori K, Weng SM, Arzberger T, May S, Rentzsch K, Kremmer E, Schmid B, Kretzschmar HA, Cruts M, Van Broeckhoven C (2013). The C9orf72 GGGGCC repeat is translated into aggregating dipeptide-repeat proteins in FTLD/ALS. Science.

[54] Ash PE, Bieniek KF, Gendron TF, Caulfield T, Lin WL, Dejesus-Hernandez M, van Blitterswijk MM, Jansen-West K, Paul JW, Rademakers R (2013). Unconventional translation of C9ORF72 GGGGCC expansion generates insoluble polypeptides specific to c9FTD/ALS. Neuron.

[55] Gendron TF, Chew J, Stankowski JN, Hayes LR, Zhang YJ, Prudencio M, Carlomagno Y, Daughrity LM, Jansen-West K, Perkerson EA (2017). Poly(GP) proteins are a useful pharmacodynamic marker for C9ORF72-associated amyotrophic lateral sclerosis. Sci Transl Med.

[56] Nguyen L, Montrasio F, Pattamatta A, Tusi SK, Bardhi O, Meyer KD, Hayes L, Nakamura K, Banez-Coronel M, Coyne A (2020). Antibody therapy targeting RAN proteins rescues C9 ALS/FTD phenotypes in C9orf72 mouse model. Neuron.

[57] Irwin KE, Jasin P, Braunstein KE, Sinha I, Bowden KD, Moghekar A, et al. A fluid biomarker reveals loss of TDP-43 splicing repression in pre-symptomatic ALS. bioRxiv. 2023 2023.2001.2023.525202.

[58] Seddighi S, Qi YA, Brown A-L, Wilkins OG, Bereda C, Belair C, et al. Mis-spliced transcripts generate *de novo* proteins in TDP-43-related ALS/FTD. bioRxiv. 2023 2023.2001.2023.525149. 10.1126/scitranslmed.adg7162 PMC11325748 38277467

[59] Iannitelli DE, Tan A, Nguyen E, Babu A, Elorza SD, Joseph T, et al. ALS sensitive spinal motor neurons enter a degenerative downward spiral of impaired splicing and proteostasis. bioRxiv. 2022 2022.2003.2026.485939.

[60] Heo D, Ling JP, Molina-Castro GC, Langseth AJ, Waisman A, Nave KA, Mobius W, Wong PC, Bergles DE (2022). Stage-specific control of oligodendrocyte survival and morphogenesis by TDP-43. Elife.

[61] Brettschneider J, Arai K, Del Tredici K, Toledo JB, Robinson JL, Lee EB, Kuwabara S, Shibuya K, Irwin DJ, Fang L (2014). TDP-43 pathology and neuronal loss in amyotrophic lateral sclerosis spinal cord. Acta Neuropathol.

[62] Rohan Z, Matej R, Rusina R, Kovacs GG (2014). Oligodendroglial response in the spinal cord in TDP-43 proteinopathy with motor neuron involvement. Neurodegener Dis.

[63] Riva N, Gentile F, Cerri F, Gallia F, Podini P, Dina G, Falzone YM, Fazio R, Lunetta C, Calvo A (2022). Phosphorylated TDP-43 aggregates in peripheral motor nerves of patients with amyotrophic lateral sclerosis. Brain.

[64] Pattle SB, O'Shaughnessy J, Kantelberg O, Rifai OM, Pate J, Nellany K, Hays N, Arends MJ, Horrocks MH, Waldron FM, Gregory JM (2022). pTDP-43 aggregates accumulate in non-central nervous system tissues prior to symptom onset in amyotrophic lateral sclerosis: a case series linking archival surgical biopsies with clinical phenotypic data. J Pathol Clin Res.

[65] Vatsavayai SC, Yoon SJ, Gardner RC, Gendron TF, Vargas JN, Trujillo A, Pribadi M, Phillips JJ, Gaus SE, Hixson JD (2016). Timing and significance of pathological features in C9orf72 expansion-associated frontotemporal dementia. Brain.

[66] Lopez ER, Borschel WF, Traynor BJ (2022). New antisense oligonucleotide therapies reach first base in ALS. Nat Med.

[67] Guerra San Juan I, Nash LA, Smith KS, Leyton-Jaimes MF, Qian M, Klim JR, Limone F, Dorr AB, Couto A, Pintacuda G (2022). Loss of mouse Stmn2 function causes motor neuropathy. Neuron.

[68] Van Alstyne M, Tattoli I, Delestree N, Recinos Y, Workman E, Shihabuddin LS, Zhang C, Mentis GZ, Pellizzoni L (2021). Gain of toxic function by long-term AAV9-mediated SMN overexpression in the sensorimotor circuit. Nat Neurosci.

[69] Bulcha JT, Wang Y, Ma H, Tai PWL, Gao G (2021). Viral vector platforms within the gene therapy landscape. Signal Transduct Target Ther.

[70] Miller TM, Cudkowicz ME, Genge A, Shaw PJ, Sobue G, Bucelli RC, Chio A, Van Damme P, Ludolph AC, Glass JD (2022). Trial of antisense oligonucleotide Tofersen for SOD1 ALS. N Engl J Med.

[71] Biogen and Ionis Announce Topline Phase 1 Study Results of Investigational Drug in C9orf72 Amyotrophic Lateral Sclerosis [https://investors.biogen.com/news-releases/news-release-details/biogen-and-ionis-announce-topline-phase-1-study-results].

[72] Finkel RS, Mercuri E, Darras BT, Connolly AM, Kuntz NL, Kirschner J, Chiriboga CA, Saito K, Servais L, Tizzano E (2017). Nusinersen versus sham control in infantile-onset spinal muscular atrophy. N Engl J Med.

[73] Syed YY (2016). Eteplirsen: first global approval. Drugs.

[74] Eser G, Topaloglu H (2022). Current outline of exon skipping trials in Duchenne muscular dystrophy. Genes (Basel).

[75] Ito D (2022). Promise of nucleic acid therapeutics for amyotrophic lateral sclerosis. Ann Neurol.

[76] Konermann S, Lotfy P, Brideau NJ, Oki J, Shokhirev MN, Hsu PD (2018). Transcriptome engineering with RNA-targeting type VI-D CRISPR effectors. Cell.

[77] Gadgil A, Raczynska KD (2021). U7 snRNA: a tool for gene therapy. J Gene Med.

[78] Lesman D, Rodriguez Y, Rajakumar D, Wein N (2021). U7 snRNA, a small RNA with a big impact in gene therapy. Hum Gene Ther.

[79] Naryshkin NA, Weetall M, Dakka A, Narasimhan J, Zhao X, Feng Z, Ling KK, Karp GM, Qi H, Woll MG (2014). Motor neuron disease. SMN2 splicing modifiers improve motor function and longevity in mice with spinal muscular atrophy. Science.

[80] Warner KD, Hajdin CE, Weeks KM (2018). Principles for targeting RNA with drug-like small molecules. Nat Rev Drug Discov.

[81] Darras BT, Masson R, Mazurkiewicz-Beldzinska M, Rose K, Xiong H, Zanoteli E, Baranello G, Bruno C, Vlodavets D, Wang Y (2021). Risdiplam-treated infants with type 1 spinal muscular atrophy versus historical controls. N Engl J Med.

[82] Ratni H, Scalco RS, Stephan AH (2021). Risdiplam, the first approved small molecule splicing modifier drug as a blueprint for future transformative medicines. ACS Med Chem Lett.

[83] QurAlis to Present at H.C. Wainwright BioConnect 2022 Virtual Conference [https://quralis.com/quralis-presents-data-about-stathmin-2-role-in-neuronal-disease-biology-and-tdp-43-biomarker-identification-at-ad-pd-2022/].

[84] Zhu F, Nair RR, Fisher EMC, Cunningham TJ (1845). Humanising the mouse genome piece by piece. Nat Commun.

[85] The Jackson Laboratory. C57BL/6J-Stmn2em6(STMN2)Lutzy/Mmjax [https://www.jax.org/strain/034061].

